# Inhibition of Tax transformation activity using a small molecule targeting Tax/PDZ domain interactions

**DOI:** 10.1186/1742-4690-11-S1-P104

**Published:** 2014-01-07

**Authors:** Karim Blibek, Naoaki Fujii, Sebastien Legros, Mathieu Boxus, Jean-François Dewulf, Pascale Zimmermann, Richard Kettmann, Franck Dequiedt, Jean-Claude Twizere

**Affiliations:** 1 Interdisciplinary Cluster for Applied Genoproteomics (GIGA), University of Liège, Liège, Belgium; 2 Department of Chemical Biology and Therapeutics, St. Jude Children's Research Hospital, Memphis, Tennessee, USA; 3 Department of Human Genetics, Faculty of Medicine, University of Leuven, Leuven, Belgium

## 

Primate T-lymphotropic virus species comprise four members (HTLV-1 to -4) that have been discovered in human. Only the HTLV-1 infection leads to adult T-cell leukemia/lymphoma (ATLL). All the four viruses share a similar genomic organization and encode transforming Tax oncoproteins. In contrast to HTLV-2 and 4, HTLV-1 and 3 Tax proteins contain a PSD-95/Drosophila Discs Large/Zona Occludens-I (PDZ) binding motif at their C-terminal that has been shown to play crucial roles in the distinct transforming properties of the Tax proteins.

Here, we used a collection of human full-length protein-coding open reading frames (ORFeome v3.1) to identify novel PDZ domain containing proteins that specifically interact with HTLV-1 Tax. Novel Tax interactors include syntenin-1 and -2, LNX2, DVL3, GIPC2, INTU, PDLIM4 and -7, RADIL and RGS3. These proteins are involved in diverse biological processes including cell division, cell fate determination, and cell survival. We further characterized interaction between Tax and syntenins and showed that, FJ9 a small molecule able to disrupt Tax/PDZ interactions, could antagonize Tax-transformation activity in a Rat-1 model.

Our study identify novel PDZ-containing proteins interacting with HTLV-1 Tax and provides the first example where Tax protein-protein interactions with PDZ containing proteins and Tax-transformation capacity could be inhibited by a small molecule.

